# Will organic thermoelectrics get hot?

**DOI:** 10.1098/rsta.2018.0352

**Published:** 2019-07-08

**Authors:** Mariano Campoy-Quiles

**Affiliations:** Institute of Materials Science of Barcelona (ICMAB-CSIC), Campus UAB, Bellaterra 08193, Spain

**Keywords:** organic thermoelectrics, polymers, carbon nanotubes, doping, anisotropy, thermal conductivity

## Abstract

The generally low energy density from most heat sources—the Sun, Earth as well as most human activities—implies that solid-state thermoelectric devices are the most versatile heat harvesters since, unlike steam engines, they can be used on a small scale and at small temperature differences. In this opinion piece, we first discuss the materials requirements for the widespread use of thermoelectrics. We argue that carbon-based materials, such as conducting polymers and carbon nanotubes, are particularly suited for large area and low-temperature operation applications, as they are abundant, low-toxicity and easy to process. We combine experimentally observed macro-trends and basic thermoelectric relations to evaluate the major performance limitations of this technology thus far and propose a number of avenues to take the thermoelectric efficiency of organic materials beyond the state of the art. First, we emphasize how charge carrier mobility, rather than charge density, is currently limiting performance, and discuss how to improve mobility by exploiting anisotropy, high persistence length materials and composites with long and well-dispersed carbon nanotubes. We also show that reducing thermal conductivity could double efficiency while reducing doping requirements. Finally, we discuss several ways in which composites could further boost performance, introducing the concept of interface engineering to produce phonon stack-electron tunnel composites.

This article is part of a discussion meeting issue ‘Energy materials for a low carbon future'.

## 1. Harvesting low-temperature heat using thermoelectrics

The transformation of heat into electricity has been established for many decades. Conversion efficiencies range from 10% to 50% for a heat source temperature varying from *ca* 400 K to 1100 K. It also depends on other factors such as the type of engine (e.g. Kalina, Rankine, Org. Rankine, Brayton, etc.) or the source of heat (e.g. solar, geothermal, nuclear, coal, etc.) [1]. Steam engines are indeed well consolidated. Unfortunately, they do not scale down well. This means that high efficiencies require very high temperatures and a typical power greater than kW_e_ [1]. In practical terms, this results in localized power generation, with heat being obtained through combustion (coal), radioactive decay (nuclear), Sun concentration or underground hot spots. Ironically, heat is one of the most ubiquitous sources of energy on Earth. The problem is that it comes in dilute forms (see below), and thus it would be more suitable for distributed generation, for which steam engines are not efficient, while other types of devices, such as thermoelectric generators, might soon be.

**Figure 1.**
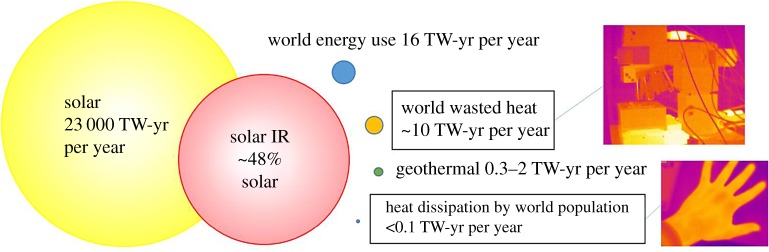
Estimated waste heat availability from different energy sources compared to the world energy use. The two pictures illustrate the wasted heat from machines and humans by showing infrared images of a piece of equipment (a Raman microscope) and the author's hand. (Online version in colour.)

There are four main sources of *natural* heat: the Sun, geothermal, waste heat from human activities and biochemical heat (body temperature). Figure 1 illustrates these sources with their approximate availabilities. By far, the most abundant source of heat is the Sun. The infrared part of the Sun spectrum alone, spanning from 800 nm to *ca* 3000 nm, already provides about 700 times more energy than the world consumes. This energy is relatively diluted (less than 500 W m^−2^), which implies that unless optical/thermal concentration is used, the heat available from the Sun will be translated in temperature differences of some tens of degrees. Geothermal heat can be divided between hot spots (like Iceland) and an average location. The former can be found at specific locations and geothermal power stations have been optimized to very efficiently harvest that source of energy. On the other hand, the heat flow from the ground at an average location is in the order of 50 mW m^−2^ [2]. This is really diluted for most practical applications. The underground temperature is, however, very stable regardless of the weather, and thus it can be used as a temperature reservoir in applications in which one side of a thermoelectric generator is buried and the other side is exposed to either atmospheric temperature or direct Sun exposure.

About 60% of world energy consumption is effectively wasted and reemitted in the form of heat. Harvesting part of the unused heat could significantly increase energy efficiency, regardless of its use as heat directly, or transformed into electricity. While domestic energy consumption typically results in low-temperature waste heat, industrial waste spans a wider range, from low to medium and even high temperatures depending on the specific industry [3]. It is also quite localized, often in accessible locations, which makes this an attractive energy source. Understandably, the power density depends strongly on the specific heat source. In transportation, combustion engines produce gases at hundreds of degrees. Part of that energy could be recovered by placing thermoelectrics in exhaust tubes. The general global trend in transportation is the shift towards an electric vehicle fleet, which would naturally reduce that heat available from combustion engines. Opportunities may then open around heat management of battery assemblies, which operate at temperatures lower than 400 K.

Lastly, assuming that all of the chemical energy consumed by humans on average (food intake) is transformed into heat, each person can be considered as a 100 W–120 W heat source spread over 1.5 to 2 m^2^ of body surface (i.e. about 50 W m^−2^, resulting in usable temperature differences below 10 degrees). Multiplied by the 7.6 billion human population leads to less than 0.1TW-yr per year. If we consider that there are about two farming mammals per human [4], the heat potential of cattle, pigs and humans together would be lower than 0.3 TW-yr per year.

Given the nature of the heat sources available, the most likely near future applications of thermoelectrics would appear to be the powering of small sensors for the internet of things, industry 4.0, agriculture 4.0 and/or wearables for sports or point-of-care therapy and diagnosis. Small sensors often have modest energy demands, in the order of hundreds of μW to a few mW [5,6]. A sufficiently high thermoelectric efficiency may render other applications viable, including solar thermoelectrics [7] or hybrid photovoltaic/thermoelectric generators [8–10], as well as heat management in buildings and electric vehicles. Generally speaking, these applications would operate at relatively low temperatures and small temperature differences.

In terms of the thermoelectric materials, the aforementioned applications would require materials performing well around room temperature. The goodness of the thermoelectric materials is quantified using the figure of merit

1.1 $$zT=\frac{\sigma S^{2}}{k}\cdot T,$$

where *S* is the Seebeck coefficient, *k* is the thermal conductivity, *σ* the electrical conductivity and *T* the average temperature. Good performing materials should have *zT* > 1 at *T* ≈ 300 K. Since the heat power density is generally small, the generators may need to have a large area to satisfy energy needs and/or be low-cost. Correspondingly, materials should be based on elements that are abundant on the Earth's crust, amenable to processing at low-thermal budget, and exhibit low toxicity. For some of the applications, such as wearables, low weight and partial flexibility would be desired.

**Figure 2.**
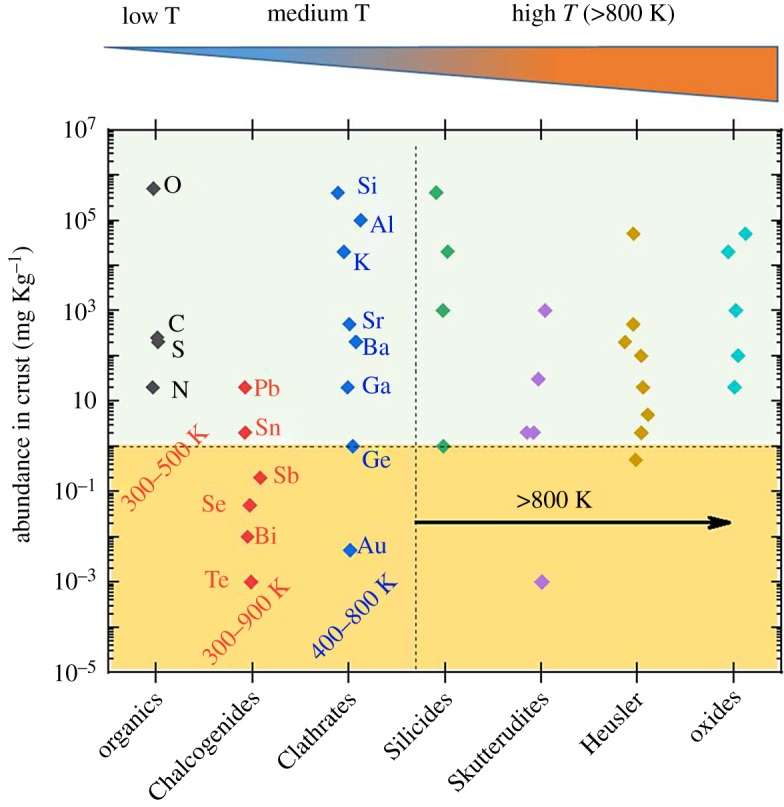
Abundance in the Earth's crust of different elements grouped by thermoelectric technologies and ordered by their corresponding optimum operation temperature range. Choice of elements in each technology follows reference [11]. (Online version in colour.)

Figure 2 compares the abundance of the elements often used in different thermoelectric technologies as a function of the temperature range in which they operate best. For operation temperatures above 800 K there are several technologies that have shown promising performance and simultaneously are based on abundant non-toxic materials [11]. For operation temperatures below 500 K, the situation is not so clear. The commercially available room temperature technology is based on chalcogenides, a class of material that performs well but is based on non-abundant elements (figure 2), often toxic (e.g. Pb), and moreover, elements that are heavy and mechanically rigid.

Carbon-based materials, such as conducting polymers and carbon nanotubes, are abundant, low toxicity, light weight and mechanically flexible materials (figure 4*a*), well-suited for low-temperature operation. Performance is encouraging but still moderate, with *zT* values lower than 0.5 for both polymers and carbon nanomaterials (nanotubes – CNT, fullerenes and graphene) [11]. Thermal and environmental stability are also concerns, especially for n-type thermoelectrics. Given the solid potential of organic-based materials for sustainable thermoelectric applications, it would be desirable to understand what is currently limiting their performance and devise strategies to move forwards.

## 2. Limiting factors in the performance of organic thermoelectrics

**Figure 3.**
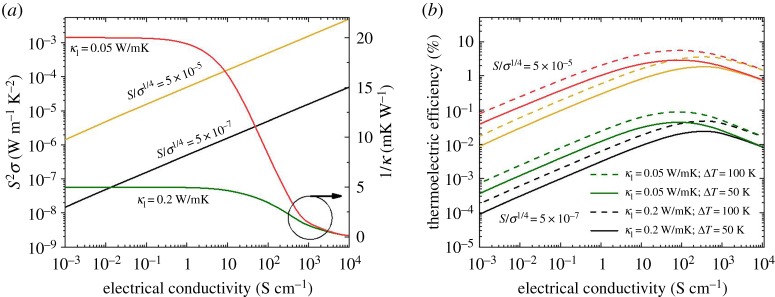
Calculated power factor (*a*, left axis), thermal conductivity (*a*, right axis) and thermoelectric efficiency (*b*) as a function of electrical conductivity. Black line (S/σ^1/4^ = 5 × 10^−7^) makes use of the proportionality factor between S and σ that is found in average for the large datasets included in reference [12], while the yellow line (S/σ^1/4^ = 5 × 10^−5^) represents the maximum values found in the literature thus far. In (*b*), solid lines (dashed) represent values for a temperature difference of 50 K (100 K). (Online version in colour.)

In a very enlightening piece of work, Chabynic, Segalman *et al.* discovered that the Seebeck coefficient for a large number of organic thermoelectric materials was proportional to *σ*^−1/4^, and thus the power factor (*S*^2^*σ*) increased with *σ*^1/2^ [12] (figure 3*a*). The fact that this empirical rule was satisfied by many systems with very different chemical structures prompted strong research activities in two directions. On the one hand, the most standard theories did not predict this behaviour, so more advanced fundamental theories of electronic transport were advanced [13–15]. On the other hand, it suggested that further improvement in power factor could come as a result of further increases in the electrical conductivity: no maximum has been yet detected in the S^2^*σ* versus conductivity for organic-based compounds.

**Figure 4.**
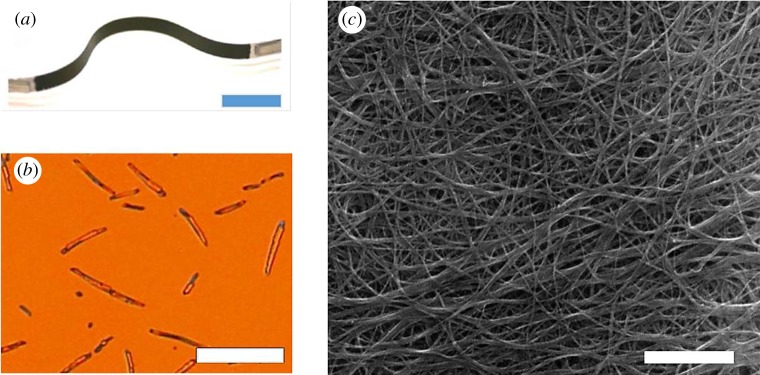
(*a*) Photograph of a polythiophene/CNT film on PET substrate (scale bar 10 mm). (*b*) Microscope image of a P3HT sample vapour doped with F4TCNQ, in which dopant crystals are clearly visible on the surface of the film (scale bar 100 µm). (*c*) SEM image of a typical CNT bucky paper (scale bar 500 nm). (Online version in colour.)

A large body of work has since been produced with the aim of improving doping both for polymers [16–19] and CNTs [20,21]. The standard doping method, namely molecular doping, has a number of challenges, including controlling the ratio between partial and integer charge transfer, phase separation of the semiconductor and the dopant (e.g. figure 4*b*), vitrification of the polymer which results in loss of the mechanical properties and difficulty to dope thick films, and temperature-assisted de-doping. A number of interesting approaches have been proposed to address these issues. For instance, performing the doping step after the film has been formed ensures good polymer microstructure and thus undisturbed charge carrier mobility [18]. Substituting the standard alkyl chains by polar sidechains has also been proved to be an efficient way of increasing compatibility with the dopants and enhancing doping efficiency while retaining good mechanical properties [17,22–24]. Multicomponent systems containing flexible polymer matrices could also help to retain flexibility [25,26]. To address the technological issue of doping thick films, a number of methods have been proposed, such as multilayer formation (which takes advantage of the fact that doped films are insoluble [27,28]), or introducing porosity [29]. Carbon nanotube matts and composites are naturally very porous and thus dopants can easily go through the films (figure 4*c* and reference [30]). The challenge there is to be able to produce the thick film itself, as CNTs do not readily dissolve in common solvents.

An additional difficulty arises from the fact that polymers and CNTs are intrinsically anisotropic structures, which likely results in anisotropic thermoelectric properties [31–33]. The Seebeck coefficient and electrical conductivities are typically measured in thin films in the direction parallel to the surface of the film. Standard techniques to measure thermal conductivity, such as 3-omega, give out-of-plane values, and thus ZT values cannot be consistently given. The development of accurate techniques for anisotropic measurements represents an ongoing issue in the field.

In summary, most recent efforts have been focused on understanding and controlling doping level and the microstructure of the materials.

## 3. Going beyond the state of the art in organic thermoelectrics

The original work where the proportionality of *S*^2^*σ* with *σ*^1/2^ was disclosed showed large scattering of the experimental data. The spreading was justified as variations in charge carrier mobility (*μ*). While the Seebeck coefficient decreases with the charge density (*n*), the electrical conductivity depends on both *μ* and *n*, *σ *= eμn. Or in other words, for a given *n*, increasing mobility would leave *S* unaffected.

In figure 3*a*, the power factors for two proportionality constants are shown, namely the one that is calculated using the average experimental relationship (black line) and a factor that would pass through the best experimental points reported so far (yellow). The performance varies over two orders of magnitude, which strongly suggests that mobility is a key parameter for thermoelectrics, so far not purposely targeted in detail. The large fraction of dopants required to dope organic semiconductors often results in a reduction in charge carrier mobility by, among other factors, disturbing morphology [18,34] and changing the density of states [35]. Therefore, an in-depth understanding of the doping process, and in particular of doping efficiency, will be very valuable in order to maintain the number of dopants as low as possible while keeping electrical conductivity high. Recent breakthroughs in this direction include the understanding of the dynamics of the integer charge transfer [36] and the possibility of making each dopant contribute with more than one charge carrier [37].

If we look at the complete figure of merit, the thermal conductivity should also be considered. Generally speaking, polymers containing some fraction of amorphous domains have very low lattice thermal conductivities [32,38]. However, the total thermal conductivity is the sum of the lattice and electronic thermal conductivities, *k* *=* *k_l_* *+* *k_e_*. The electronic thermal conductivity is related to the electrical conductivity by the Wiedemann-Franz Law, *k_e_* = (*k_B_e*)2*LσT*, where *k*_B_ is the Boltzmann constant, and *L* the dimensionless Lorenz factor. If, for simplicity, we assume the theoretical Sommerfeld value for a degenerate Fermi gas, *L* = π^2^/3, the total thermal conductivity can be estimated for a given *k_l_*. Figure 3*a* shows 1/*k* for two cases, a typical value of *k_l_* = 0.2 Wm^−1^ K^−1^, and the very low value reported for fullerenes *k_l_* = 0.05 Wm^−1^ K^−1^ [39]. Clearly, the potential advantage of low thermal conductivity of polymers is in part lost for conductivities above around 1000 S cm^−1^.

The thermoelectric efficiency at an average temperature *T_m_*, is given by:

3.1 $$\eta =\frac{T_{\text{hot}-}T_{\text{cold}}}{T_{\text{hot}}}\cdot \frac{\sqrt{1+ZT_{m}}-1}{\sqrt{1+ZT_{m}}+T_{\text{cold}}\text{/}T_{\text{hot}}}.$$

Figure 3*b* shows *η* as a function of electrical conductivity for several materials scenarios. The first observation is that a maximum is observed. This arises from the compensation of electrical and thermal properties. This observation is very important as it suggests that the degree of doping already achieved is good enough for the materials used. Efforts should be focused, then, not so much on further increasing doping (i.e. the charge density) but rather on mobility, as this would produce a much higher efficiency, as shown in figure 3*b*. Clearly, research efforts to investigate polymers with high mobilities—such those developed for transistor applications that are based on polymers with high persistence length that form basically amorphous films [40,41]—and control their doping level would be an interesting avenue towards high efficiencies. For a given material system, known factors affecting charge carrier mobility, such as molecular weight [42] and molecular orientation [33] should be explored as a good path towards decoupling *S* and *σ*.

On the other hand, CNTs have a much higher intrinsic *μ* compared to polymers, and in this respect, the proportionality constant between power factor and conductivity is expected to be higher, as it has been shown [43]. Well-dispersed CNTs, perhaps with the help of wrapping polymers [30,44,45], will result in better mobility values [46], as would be expected also for long CNTs.

Figure 3*b* evaluates another two avenues for increasing organic thermoelectric performance. An obvious one is to operate at slightly higher temperature differences. From the simple estimates here one can see that doubling Δ*T* would also result approximately in a factor of 2 increase in thermoelectric efficiency. The judicious choice of thermally resistant polymer and CNT has been recently shown to perform at 500 K even better than at room temperature, due to thermally assisted conductivity [30].

Besides mobility and temperature difference, another factor often overlooked is the thermal conductivity. In the case of CNTs, making composites is precisely aimed at reducing thermal conductivity [11,47–49]. However, in the case of polymers, no particular action is typically taken despite the foreseeable problem coming from the aforementioned rising electronic contribution to the thermal conductivity upon doping (figure 3*a*). It is interesting to think of how a further reduction in lattice thermal conductivity would influence the performance. Figure 3 shows that the thermoelectric efficiency would double if the lattice thermal conductivity decreased from the typical value of *k_l_* = 0.2 Wm^−1^ K^−1^, down to the value reported for fullerenes *k_l_* = 0.05 Wm^−1^ K^−1^, while maintaining the electronic contribution from the Wiedemann-Franz Law. Clearly, a fine control of the lattice thermal conductivity could boost efficiency. Interestingly, reducing lattice thermal conductivity shifts the efficiency maximum to lower electrical conductivities, with the concomitant relaxation of doping requirements. Adding fullerenes to polymers in order to produce rattling-like molecules [39,50] that scatter phonons might be a method to reduce *k_l_*.

Alternatively, decoupling *σ* and *k* might also be possible by exploiting the intrinsic anisotropic properties of most organic materials. Both thermal and electronic conductivities are higher along the polymer backbone (or CNT axis) compared to interchain (intertube) values. Extremely highly oriented polymers can have thermal conductivities along the chain as high as 100 Wm^−1^ K^−1^ [51,52]. In most cases, however, thermal anisotropy is only a factor of 2–3 [31], while the electron mobility anisotropy can be more than one order of magnitude [53,54]. The ratio of the two would, then, also be close to an order of magnitude or more. For a description of orientation methods in semiconducting polymers, the reader is referred to recent reviews such as reference [55].

## 4. Mixing and interfaces

**Figure 5.**
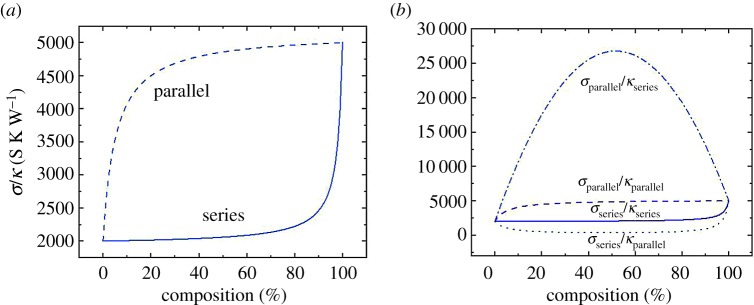
Calculated ratio of the electrical to thermal effective conductivity of a composite as a function of composition for parallel (dashed line), series (solid line) connection between components (*a*). In (*b*), we show the same ratio for a hypothetical case in which the two materials are connected electrically in series and thermally in parallel (dotted line) or electrically in parallel and thermally in series (dash-dotted line). The latter case might be found for a selective interface which scatters phonons strongly but lets electrons go through, referred to in the text as thermally stacked-electrically tunnelled composites. (Online version in colour.)

What is the thermoelectric performance of a composite? If we use the rule of mixtures for the electrical and thermal conductivities, the performance of the composite will always be in between those of the pristine materials (figure 5*a*). This happens no matter whether the materials are connected in series or in parallel since the equations governing the effective conductivity values are:

4.1 $$\sigma_{\text{parallel}}=v_{1}\cdot \sigma_{1}+(1-v_{1})\cdot \sigma_{2}$$

and

4.2 $$\frac{1}{\sigma_{\text{series}}}=\frac{v_{1}}{\sigma_{1}}+\frac{1-v_{1}}{\sigma_{2}},$$

where *σ_i_* and *v_i_* are the conductivity and volume fraction of component *i*, respectively. Similar equations apply for the thermal conductivity. Figure 5*a* shows the ratio *σ*/*k*, as this would be the factor that enters the figure of merit (equation (1.1)). Note that *S* will be the sum of the individual *S* weighted by the volume and by the electrical conductivity, and thus it is often dominated by the *S* of the material with higher *σ* already at low concentrations (see also below).

More elaborated theories would also predict that composites do not outperform single materials [56]. Mixtures of two materials, however, may not be well described by effective medium models if interfaces play a strong role. Let us do a *gedanken* experiment. Let us imagine that phonons and electrons are scattered differently at the interfaces in such a way that the electrons can quantum tunnel through the interphase while there is a strong thermal impedance mismatch between the two materials that reduces the rate of heat transfer between components. This might be, for instance, the case of a percolating system of CNTs with a very thin wrapping polymer layer placed at the interfaces which a charge carrier can tunnel through. (Note that tunnelling would depend on the energy barrier properties rather than on the polymer mobility as would be the case in a series connected composite). This scenario is equivalent to having a parallel connection for electrons and a series connection for the phonons. Then, the ratio of the electrical conductivity to the (lattice) thermal conductivity of the composite will show a maximum which can be much higher than any of the pristine components (figure 5*b*). Conversely, if the thermal transport was parallel and the electrical transport in series, the composites would perform much worse than either component. The synergetic effect comes when, at thermoelectric leg level, the material behaves in parallel for electronic transport and in series for thermal transport, which is exactly the opposite of the connection between legs in a typical thermoelectric generator (electrically in series and thermally in parallel).

This ‘selective interface' effect, i.e. phonon stack-electron tunnel composites, might be happening to a certain degree in some reported systems. For instance, there are different reports of polymer/CNT composites that simultaneously show the high *σ* of the CNTs and the low *k* of the polymer (e.g. [30,47,48]). Layer-by-layer systems combining polymers, graphene and CNTs might also be partially acting like this [57,58]. The increase in Seebeck coefficient as a function of number of layers observed on that system might suggest energy filtering effects too. Understanding of the role of interfaces in organic thermoelectrics is still in its infancy, but it is clearly a very promising route towards designing composites with outstanding performances.

Besides interfaces, mixing two materials may result in modification of the bulk electronic properties of the composite. In a series of papers, Kemerink *et al.* demonstrated that the density of states can be engineered by blending two different polymers [14,34,59]. If the materials are chosen with the right energy levels and in particular weight ratios, the energies associated with the charge transport and Fermi levels can be separated, yielding a huge increase in the Seebeck coefficient. While the power factor is not improved due to the concomitant reduction in *σ* by blending, these reports clearly demonstrate that manipulation of the density of states might also be a valuable path for going beyond the state of the art.

## 5. Broader scope opportunities

The power factor versus *σ* plot demonstrated that meta-studies incorporating large data volumes can lead to a deeper understanding of the materials. Going a step forward in this bearing, we believe that high throughput screening and data science methodologies will also play a role in the development of organic thermoelectrics. This is already becoming a reality in organic photovoltaics, where combinatorial screening [60,61] and machine learning algorithms [62–64] are being applied.

Stability is also a key issue that affects all organic related devices. The fact that organic light emitting based TV screens became commercially available a few years ago suggests that there is not a fundamental limitation that would prevent real-world application of this class of materials. However, important issues still need to be understood and addressed depending on each technology and its requirements (e.g. in terms of encapsulation and operation conditions). In the case of organic thermoelectrics, perhaps one pressing need is to obtain stable doping levels, particularly stable n-type materials. Stability here should be checked upon thermal stress, as two of the main concerns are thermally assisted de-doping and oxidation.

Another area that may soon become important is the assessment of the different organic thermoelectric categories through life cycle analysis (LCA) methodologies. While a somewhat preliminary study has already been published for PEDOT-based devices [65], there is plenty of room for researchers to work on this field. Besides addressing the energy requirements for fabrication, energy payback times and other aspects, LCA could help guide experimentalists with respect to the device geometry. While in solar cells the geometry is almost invariably the same (a planar stack containing a substrate, two electrodes, two blocking layers and the active layer), this is not the case for thermoelectrics. This is because while in photovoltaics the source of energy is typically the same, i.e. the Sun (except for indoor applications), the sources of heat are diverse. The geometry of standard inorganic thermoelectric generators is not necessarily the best for all applications and materials. For instance, solution processing of materials and a flexible generator design may strongly alleviate the high fraction of the TE cost associated with heat exchangers in typical inorganic TE. LCA may prove very helpful in the design of the best organic based thermoelectric generators for low-temperature applications. Aspects such as compatibility with roll-to-roll deposition [6,65], thermoelectric leg dimensions [5], generator shape [66,67], recyclability [30], economics [68], etc. might all be LCA evaluated.

## 6. Conclusion

Most of the available, unused heat produced in nature or through human activities is diluted and low temperature. The good scalability of thermoelectric generators makes them particularly suitable for harvesting this heat and producing electricity. If thermoelectrics are to have some impact, a technology should be developed that is based on abundant, non-toxic materials deposited through easy and low-thermal-budget processing techniques, and materials that simultaneously exhibit high efficiency and good stability. Carbon-based thermoelectrics could be that technology. In this opinion piece, different avenues towards competitive performance have been highlighted, including the potential of improvements in charge carrier mobility, introducing molecular orientation, further reducing thermal conductivity, and designing phonon stack-electron tunnel composites.

## Data accessibility

This article has no additional data.
